# 放射性^125^Ⅰ粒子植入治疗纵隔内恶性肿瘤及淋巴结转移癌43例分析

**DOI:** 10.3779/j.issn.1009-3419.2011.12.06

**Published:** 2011-12-20

**Authors:** 凌飞 罗, 洪武 王, 洪明 马, 存良 蔡, 洁莉 张, 珩 邹

**Affiliations:** 100028 北京，煤炭总医院肿瘤微创治疗中心 Minimal Invasive Tumor Therapy Center, Meitan General Hospital, Beijing 100028, China

**Keywords:** ^125^Ⅰ, 粒子, 植入, 纵隔内, 恶性肿瘤, 放射治疗, ^125^Ⅰ, Seed, Implantation, Mediastinal, Malignant tumor, Brachytherapy

## Abstract

**背景与目的:**

纵隔内恶性肿瘤位置深邃、隐匿, 手术切除可能性极小。由于其紧邻心包、主气管、大血管等重要解剖结构, 氩氦刀冷冻及热消融同样受到限制。本文比较43例放射性^125^Ⅰ粒子植入患者的影像复查资料和生存质量, 探讨其操作安全性和临床疗效。

**方法:**

2010年7月-2011年7月经病理证实并完成随访的43例患者, 其中原发纵隔型肺鳞癌21例, 原发食管癌9例, 淋巴结转移癌13例。合并主气道50%以上狭窄18例, 食管梗阻9例, 上腔静脉回流障碍6例。每个病灶植入^125^Ⅰ粒子10枚-60枚, 平均(30.79±14.23)枚。治疗后2个月、4个月、6个月及12个月复查CT观察局部病灶控制情况, 并对患者生存质量及生存期进行随访。

**结果:**

全部操作技术成功率100%, 最长随访时间12个月, 6个月生存37例, 6个月生存率为85.0%, 局部病灶PR 30个, NC 7个, 临床有效率为81.08%, 临床受益率为100%。12个月生存31例, 1年生存率为60.5%, 局部病灶CR 16个, PR 7个, NC 2个, PD 6个, 临床有效率为74.19%, 临床受益率为80.65%。6个月、12个月KPS评分提高且差异明显。穿刺导致气胸3例(6.98%), 无大血管、主气管、喉返神经及心包损伤等严重并发症。

**结论:**

放射性^125^Ⅰ粒子植入治疗纵隔内恶性肿瘤, 技术成功率高且相对安全, 临床疗效肯定。

## Abstract (en)

**Background and objective:**

The locations of mediastinal malignant tumor lesions are deep and occult, and are close to the pericardium, trachea, or major vessels.Therefore, the possibility of surgical resection is slim, and cryoablation and thermal ablation are restricted.In current study, image and life quality data were compared before and after ^125^Ⅰ seeding therapy to investigate its safety and clinical effects.

**Methods:**

From July 2010 to July 2011, a 43-patient follow-up of pathologically confirmed cancers, including 21 cases of primary mediastinal squamous lung cancer, 9 cases of primary esophagus cancer, and 13 cases of lymph node metastases were completed.Among these, 18 cases presented with tracheal stenosis > 50%, 9 cases had esophageal obstruction, and 9 cases had superior vena cava reflux disorder.Each lesion was implanted with 10 to 60 pieces of ^125^Ⅰ particles, with an average of 30.79±14.23.CT data at 2, 4, 6, and 12 months after therapy were obtained to evaluate the local lesion outcome.The quality of life of the patients as well as survival data was also recorded.

**Results:**

The overall success rate of the operation was 100%.The longest time of follow-up was 12 months.At 6 months, 37 patients were alive, and the half-year survival rate was 85.0%.In terms of local lesions, 30 cases of PR and 7 cases of NC were found.The clinical effective rate was 81.08%, and the clinical beneficial rate was 100%.At 12 months after therapy, 31 patients were alive, and the one-year survival rate was 60.5%.In terms of local lesions, 16 cases of CR, 7 cases of PR, 2 cases of NC, and 6 cases of PD were found.The clinical effective rate was 74.19%, and the clinical beneficial rate was 80.65%.The KPS score increased after the treatment (*P*=0.000).Three cases of pneumothorax presented after treatment, and no severe complications, such as vessel, trachea, recurrent laryngeal nerve, or pericardiocentesis injuries, were found.

**Conclusion:**

Radiation seed implantation in mediastinal malignant tumors is a relatively safe technique with high success rate, considerable efficacy, and clear clinical value in advanced cancer treatment.

纵隔内原发性肿瘤主要包括胸腺瘤、恶性淋巴瘤、畸胎瘤、神经源性肿瘤以及纵隔型肺癌、食管癌, 继发性肿瘤主要指淋巴结转移癌。恶性淋巴瘤全身化疗效果满意, 神经源性肿瘤以良性占多数。纵隔型肺癌、食管癌和淋巴结转移癌位置深邃、隐匿, 多数失去手术切除机会且对全身化疗不敏感。放射性粒子植入治疗恶性肿瘤已有100多年历史^[1]^, 近距离治疗效果明显, 已成为一种新的微创治疗手段。本文对43例患者治疗前后的影像资料和生存质量进行总结, 以探讨放射性^125^Ⅰ粒子植入治疗纵隔内恶性肿瘤的操作安全性和临床疗效。

## 1 资料与方法

### 1.1 患者资料

2010年7月-2011年7月经病理证实并完成随访的43例患者, 共43个病灶。包括男性32例, 女性11例, 年龄16岁-90岁, 平均年龄(58.07±13.04)岁。原发纵隔型肺鳞癌21例, 临床TNM分期Ⅲb期13例, Ⅳ期8例。原发食管癌8例, 食管癌术后复发1例, 临床TNM分期Ⅲb期5例, Ⅳ期4例。淋巴结转移癌13个, 压迫主气管或上腔静脉出现明显的通气或静脉回流障碍。主气道50%以上狭窄18例, 气促指数4级11例, 5级7例, 食管梗阻9例, 上腔静脉回流障碍6例。治疗前肿瘤最大径(3.6-8.2)cm(平均5.38 cm), 患者KPS评分70分29例、60分9例、50分5例, 平均(65.58±7.00)分。

### 1.2 适应证及方法

#### 1.2.1 适应证

肿瘤病理诊断明确; 肿瘤位于纵隔内, 没有手术切除可能且KPS评分不低于50分; 增强扫描病灶CT值较平扫升高不足20 Hu, 提示乏血; 患者出现明显的临床症状, 如气促、胸痛、食管梗阻; 淋巴结转移癌压迫主气管、上腔静脉并有明确的临床症状, 如气促或颜面、上肢、脑组织静脉回流障碍。

#### 1.2.2 禁忌证

KPS评分40分以下, 不能耐受有创性操作; 严重肺、肝、肾或心功能衰竭; 有明显的出血倾向; 肺穿刺禁忌, 如肺大疱等。

#### 1.2.3 方法

全部操作在CT引导下进行, 依据病灶位置取不同体位扫描, 确定皮肤穿刺点。参照ATS纵隔内淋巴结分布概念, 5区、6区、4L、10-11R、L病灶前胸壁进针, 2R、3R、4R、7区、8区、10-11R、L病灶肩胛间区进针, 2L、3L病灶胸骨上窝进针。依计算机治疗计划系统(therapy planning system, TPS)确定放射治疗剂量, 依模拟粒子分布和等量曲线、剂量直方图确定病灶周边匹配计量不低于95%, 根据病灶大小和每枚粒子活度确定所需粒子数[病灶(长+宽+高)/3×5/每枚粒子活度]。每枚粒子活度0.6 mCi-0.8 mCi, 辐射范围1 cm^2^, 半衰期59.6天, 1 mCi产生182 Gy, 病灶照射剂量不低于60 Gy。严格无菌操作, 植入粒子间隔1.0 cm。每个病灶植入^125^Ⅰ粒子10枚-60枚, 平均(30.79±14.23)枚。

18例主气道50%以上狭窄患者先行气管内支架置入, 维持气道通气, 缓解气促。9例食管梗阻不能进食患者, 粒子植入后7天内行食管内支架置入后进食状况即刻改善。6例上腔静脉回流障碍患者中, 2例急诊行上腔静脉内支架植入, 4例常规抗凝治疗。

### 1.3 随访

治疗后2个月、4个月、6个月和12个月CT复查局部病灶控制情况, 并对患者主要临床症状缓解、KPS及生存期进行随访, 最长随访时间为12个月。主要临床症状缓解包括:完全缓解(complete response, CR)、部分缓解(partial response, PR)、无变化(no change, NC)。

## 2 结果

全部操作技术成功率100%(图 1), CT复扫所植入粒子均位于预定位置(图 2)。穿刺并发症包括气胸3例(6.98%), 经胸膜腔置管引流后均在1天-3天内治愈; 肺内少量出血23例(53.49%), 经药物对症治疗均在3天-7天内治愈。无交通性气胸、大血管、主气管、喉返神经及心包穿刺损伤等严重并发症。

**1:**
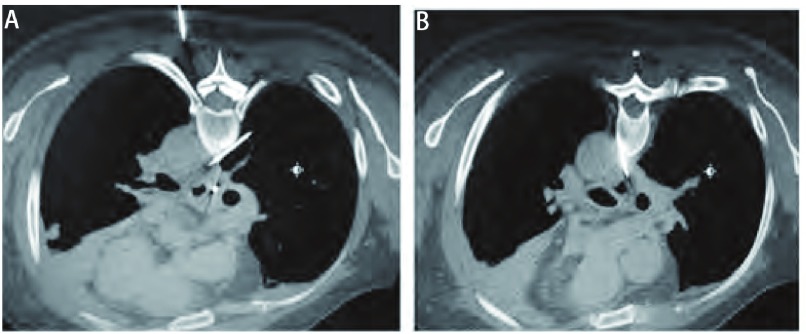
纵隔8区肿瘤。A, B:两侧肩胛间区穿刺, 穿刺针在主动脉与椎体之间安全穿过达靶灶。 Tumor in the eighth region.A, B:Puncture in the two flanks of scapular region, puncture needle reaches target region between aorta and centrum with safety.

**2:**
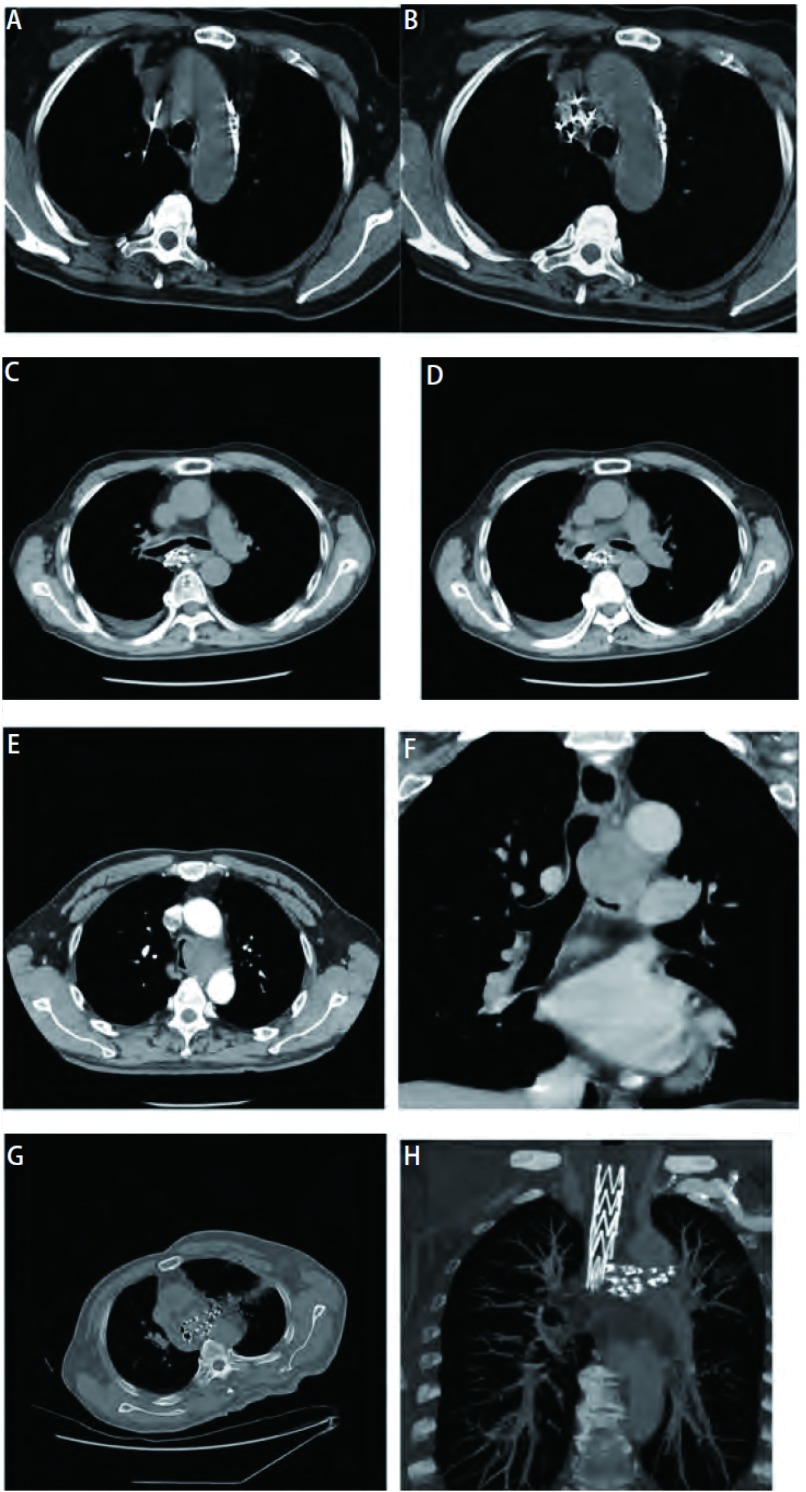
纵隔内恶性肿瘤和淋巴结转移癌。A, B:4R淋巴结转移癌, 穿刺及粒子分布; C, D:7区纵隔型肺鳞癌治疗后粒子分布; E, F:3L、4L纵隔型肺鳞癌治疗前CT增强扫描显示巨大肿瘤; G, H:与E, F同一患者, 治疗后粒子分布。 Mediastinal malignant tumors and lymph node metastases.A, B:The punctue region and particles distribution of mediastinal malignant tumor in 4R region; C, D:Particles distribution of mediastinal lung squamous cell carcinoma in 7 region; E, F:Enhanced CT scanning shows the giant tumor of mediastinal lung squamous cell carcinoma in 3L and 4L region before therapy; G, H:Particles distribution after therapy.

全部患者治疗后气道通气和进食状况即刻改善, 胸痛症状1周-2周内均有不同程度缓解。

43例全部完成随访, 2个月-12个月CT复查局部病灶情况见表 1。2个月、4个月临床受益率(CR+PR+NC)100%(图 3)。6个月生存37例(生存率为85.0%), 临床有效率(CR+PR)为81.08%, 临床受益率(CR+PR+NC)为100%, 7例肿瘤无变化, 30例肿瘤缩小 > 50%, 最大径1.2 cm-3.7 cm(平均2.46 cm), 维持稳定4周以上(图 4)。12个月生存31例(生存率为60.5%), 临床有效率为74.19%, 临床受益率为80.65%。治疗后6个月评价37例生存患者KPS为(80.00±8.16)分, 较治疗前提高且差异明显(*t*=-13.49, *P* < 0.001)。12个月评价31例生存患者KPS为(83.95±6.95)分, 较治疗前提高且差异明显(*t*=-18.47, *P* < 0.001)。

**1:** 2个月-12个月CT复查局部病灶情况 1 CT data at 2, 4, 6, and 12 months after therapy to evaluate the local lesion outcome

Efficacy	Follow-up time			
	2 months	4 months	6 months	12 months
CR	0	0	0	16
PR	0	0	30	7
NC	43	43	7	2
PD	0	0	0	0
CR:complete response;PR:partial response;NC:no change;PD:progressive disease.				

**3:**
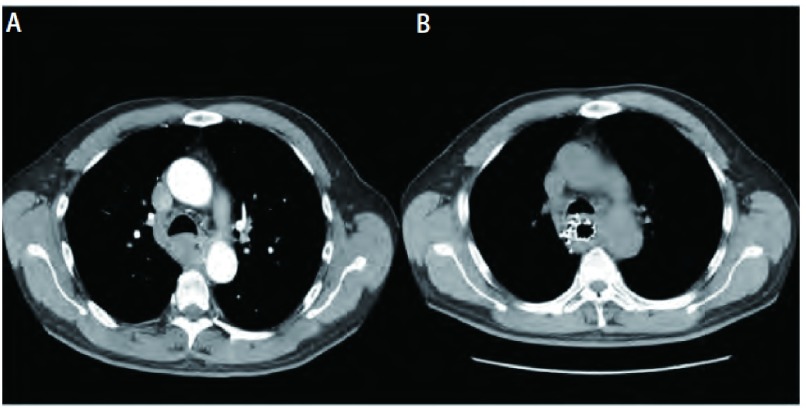
8区食管鳞癌。A:治疗前CT增强扫描显示肿瘤; B:治疗后4个月复查肿瘤NC。 Esophageal squamous cell carcinoma in the eighth region.A:Enhanced CT scanning shows the tumor before therapy; B:4 months after radioactive seeds implantation, tumor outcome is NC.NC:No change.

**4:**
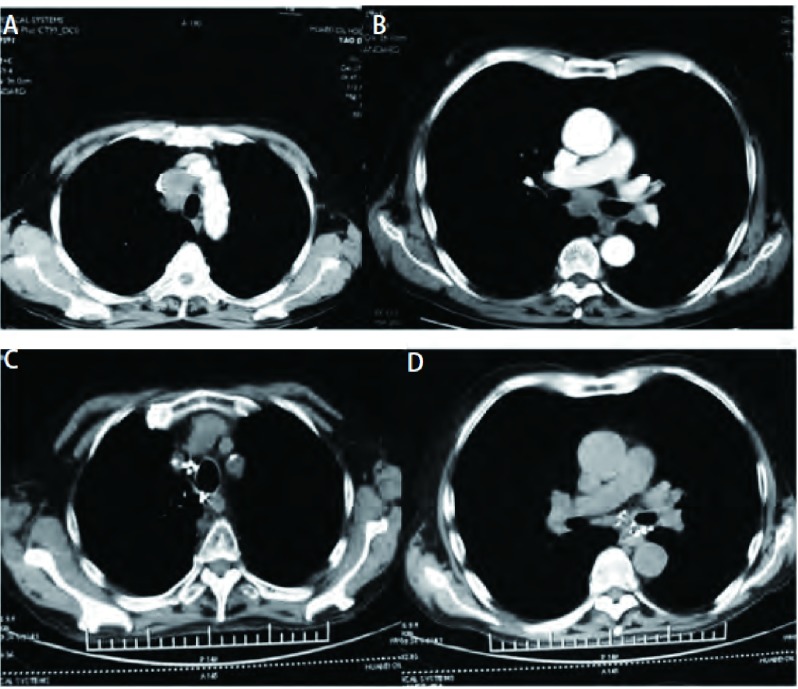
纵隔淋巴结转移癌。A, B:治疗前CT增强扫描显示4R、7区淋巴结转移癌; C, D:治疗后6个月复查病灶PR。 Lymph node metastases in mediastinum.A, B:Enhanced CT scanning shows the lymph node metastases of 4R and 7 regions before therapy; C, D:6 months after radioactive seeds implantation, tumor outcome is PR.PR:Partial response.

## 3 讨论

近年来放射性粒子植入治疗恶性肿瘤的研究不断深入, 但刚等^[2]^对^125^Ⅰ粒子治疗前后患者肿瘤标志物进行对比研究, 认为^125^Ⅰ粒子治疗肺癌能有效降低多项肿瘤标志物水平。宁厚法等^[3]^报道^125^Ⅰ粒子治疗32例肺癌的疗效, 2个月、4个月、6个月有效率分别为59.36%、83.87%和86.67%。王焕根等^[4]^报道^125^Ⅰ粒子治疗58例肺癌的疗效, 总有效率为91.4%。

由于纵隔内肿瘤位置深邃、隐匿, 紧邻心包、大血管、主气管等重要解剖结构, 故准确的穿刺是治疗成功的关键。穿刺偏差不仅使粒子分布不理想, 还往往导致心脏、大血管损伤, 甚至直接危及患者生命。我们体会4R、3L、4L和7区、8区肿瘤是穿刺难点。4R肿瘤位于上腔静脉和升主动脉后方, 前胸壁进针需穿过上腔静脉与升主动脉间的狭窄缝隙, 穿刺风险大。即便成功穿过该缝隙, 也很难进一步调整穿刺针方向, 使粒子不能按计划植于预定位置, 右侧肩胛间区进针则更为理想。3L、4L肿瘤位于主动脉弓下、肺动脉干上方, 左侧前胸壁进针是唯一选择, 除需避免损伤心脏、大血管以外, 还应避免损伤主动脉弓下走行的喉返神经, 喉返神经损伤会导致声带功能丧失, 轻者失声, 严重者进食、进水障碍。7区、8区肿瘤位于气管隆突下和食管旁, 右侧肩胛间区进针可覆盖大部分肿瘤, 但椎体、食管之间往往残留死角, 此时需经左侧肩胛间区穿刺。对于大部分患者, 如果椎体增生、动脉硬化不严重, 此路径并非禁区, 需注意紧贴椎体进针, 针尖斜面朝向主动脉相对安全。穿刺切忌一步到位, 应循序进针并及时调整进针方向。前胸壁和肩胛间区进针, 穿刺路径长, 肺内出血机率高, 经药物治疗多可痊愈。

计算机治疗计划系统最早是针对前列腺肿瘤的放射性粒子植入治疗设计完成的, 确定肿瘤放射治疗剂量、周边匹配剂量和所需粒子数后, 经会阴入路可完成多针穿刺、均匀布针。纵隔内肿瘤不同于前列腺肿瘤:首先纵隔内肿瘤位置深邃、隐匿, 允许的穿刺路径往往是异常狭小的缝隙; 此外还要穿过相当多的肺组织, 根本不允许多针同时穿刺, 更无法做到均匀布针。我们在实际操作中多采用单针穿刺, 首针穿刺成功后即可根据CT扫描图像和瘤体大小部分退针, 利用针尾调整后续进针方向, 使粒子满意分布。就穿刺安全性而言, 单针穿刺对脏层胸膜损伤最小, 气胸发生率低。需调整进针方向时, 依据CT引导部分退针, 而不是将针完全退至纵隔以外, 可避免损伤正常结构。陈明等^[5]^报道^125^Ⅰ粒子治疗30例肿瘤的疗效, 结果显示CT引导安全可靠。本组患者3例气胸, 23例肺内少量出血, 均在1周内治愈, 无心包、大血管穿刺损伤等严重并发症。

纵隔型肺癌、食管癌多为鳞癌或腺癌, 对全身化疗不敏感。肿瘤位置深邃、周边重要结构丰富, 氩氦刀冷冻、热消融等均受到明显限制。体外放疗由于放射剂量逐层衰减, 使靶灶很难获得60 Gy以上的有效治疗剂量。若想获得理想治疗效果, 必须加大初始剂量, 放射并发症则难以避免, 如放射性皮肤损伤、放射性肺炎以及食管、气管瘘等。2010年7月-2011年7月我科先后收治21例外放疗后并发食管、气管瘘患者, 由于不能进食、进水, 患者生存质量和营养状况明显降低。放射性粒子植入属近距离放疗, 为后装式插值技术, 靶区剂量均匀, 对周边正常组织损伤小, 治疗效果确切, 已成为纵隔内恶性肿瘤的重要治疗手段^[6]^。内支架成形是关键治疗技术, 可即刻改善气道通气, 改善进食状况, 再通腔静脉回流, 使患者获得宝贵的治疗机会, 但其对肿瘤本身无治疗作用, 内支架成形与粒子植入相结合可明显提高疗效。本组患者未联合全身化疗等其它治疗, 2周内临床症状得到明显改善, 18例主气道50%以上狭窄患者, 粒子植入后1个月-4个月先后取出支架, 气道通气功能良好。6个月生存率为85.0%, 局部病灶无进展。12个月生存率为60.5%, 局部病灶缩小或稳定25个, 肿瘤进展6个, CT显示均为肿瘤边缘生长, 经再次植入粒子得到控制。治疗后6个月、12个月评价生存患者KPS, 较治疗前均有明显提高且差异明显。

综上, 我们认为CT引导下放射性粒子植入治疗纵隔内恶性肿瘤或纵隔内淋巴结转移癌, 技术成功率高且相对安全, 疗效肯定, 应成为首选治疗技术。放射性粒子植入与内支架成形技术联合应用将使更多患者获得治疗机会。
